# How Do Older People Experience Person-Centred Integrated Care? An Integrative Review of the Evidence

**DOI:** 10.5334/ijic.9066

**Published:** 2025-12-15

**Authors:** Sarah Murphy, Tanya McCance, P. J. White

**Affiliations:** 1 PhD Researcher, Atlantic Technological University, IE; 2 The Mona Grey Professor of Nursing Research and Development, Ulster University, UK; 3 Senior Lecturer, South East Technological University, IE

**Keywords:** Older people, integrated care, person-centred care, experiences of care

## Abstract

**Background:**

‘Person-centred integrated care’ (PCIC) emerged in literature, policy and practice to meet the increasing care needs of an older population living longer with increased levels of chronic illness, multimorbidity and at enhanced risk of care fragmentation. Most evaluations of PCIC have been service-centred, rather than person-centred, and there is a lack of research on the effects of integrated care on patients, especially older people.

**Methods:**

This integrative review explored evidence regarding older people’s PCIC experiences, synthesising empirical literature from five databases: Medline, PsycInfo, CINAHL, Embase and Web of Science.

**Results:**

Findings included: i) definitions and components of integrated care and conceptualisations of person-centredness in the context of integrated care; ii) older people’s positive PCIC experiences featured: coordination; continuity and relational care; involvement in care, including effective communication and information about care; and holistic care; iii) integrated care optimises care when successfully delivered, however, older people’s experiences were mixed; and iv) barriers included a lack of integrated care frameworks developed from patients’ perspectives, poor communication and information and staff shortages and turnover leading to discontinuity, limited time for meaningful interactions and follow-up care.

**Conclusion:**

While PCIC optimises care experiences, its evaluation is challenged by multiple conceptualisations and lack of engagement with service users.

## Introduction

Person-centred integrated care (PCIC) models are growing in prevalence in both policy and practice to meet the increasing care needs of a growing older population that is both living longer and with increased levels of chronic illness and multimorbidity. However, despite the desirability of the PCIC approach, progress has been slow, attributed to inconsistent implementation and lack of organisational change necessary to facilitate positive PCIC service user experiences, including for older people [1,2].

There is no single accepted definition for integrated care and multiple definitions exist, varying according to sector, discipline, context and perspective [3,4,5,6,7,8,9]. Rather than a distinct concept, integrated care is often understood as an overarching and organising principle or strategy [7,10]. At its simplest, integrated care entails the integration of a fragmented health system, to optimise care and related experiences [6, p. 1]. Integrated care is characterised by complexity and in addition to its multitude of definitions, there exist many types and ways that it can manifest in practice [5,6,11]. Leutz (1999) identified three types: linkage, coordination or full integration [12]. Integration of care can happen at different levels, including micro (clinical), meso (professional and organisational) and macro (policy) levels [4,13,14,15,16]. Integration can be normative, referring to shared mission, norms and values, or functional, referring to tasks, processes of delivery and related resources with both considered vital to ensuring connectivity across levels [15,17,18]. Integrated strategies may vary in terms of their breadth and degree, relating to their reach and effect [4]. The breadth of integration can refer to whether it occurs in horizontal directions, linking similar levels of care, or vertical, linking different levels of care [6,15].

Person-centeredness is considered a core element of integrated care and quality care [19]. Similar to integrated care, PCIC is characterised by a lack of consensus regarding definition and varied terminology [20]. The emergence of PCIC and its rationale are linked with the drive to improve care experiences and prioritise the person in integrated care planning and delivery. Person-centredness is central to the majority of integrated care definitions which reference person-centred goals, values and aspirations [6,7,21,22]. It is possible for care to be integrated, from systems, service provider, funding and governance perspectives, without being person-centred from the perspectives of service users and carers. Person-centred and people-centred approaches to defining integrated care are preferred because they foreground the service user perspective and experience rather than structural or organisational aspects of care [6,7,21].

Person-centredness is conceptualised as a corrective to scientific and technological advances which have resulted in overly medicalised and dehumanising models of care, focused on system efficiencies rather than centred around the holistic needs and values of the person. It is also a policy imperative focused on patients’ rights and autonomy [23,24]. This review applied the Person-centred Practice Framework (PCPF) [24] as a sensitising tool for analysing person-centeredness. The PCPF is an internationally recognised theoretical framework which supports interprofessional teams to understand the dimensions of person-centredness and how they can be realised in practice [24]. It has been translated into ten languages and tested in multiple healthcare contexts in over 22 countries, including with older people [24,25].

Evaluations of PCIC have tended to be service-centred in terms of their focus on outcomes and assessment of successful practice, rather than person-centred [1,26]. There is a marked lack of research on the effects of integrated care on service users [27], including older adults. There is also a recognised knowledge gap regarding PCIC experiences. This knowledge gap exists across population groups but particularly for older people [26]. For these reasons, this integrative review sought to identify and synthesise available evidence on older people’s PCIC experiences. Its research question asked what empirical literature was available on the PCIC experiences of older people. Specifically the research objectives of this review were: i) to ascertain how empirical literature defines integrated care and conceptualises person-centredness in the context of integrated care; ii) to explore how PCIC impacts on older people’s care experiences; iii) to identify the main components of older people’s positive PCIC experiences; and iv) to identify the barriers to the successful provision of PCIC for older people.

A search of Medline, the Cochrane Database of Systematic Reviews and the Joanna Briggs Institute (JBI) Evidence Synthesis was conducted and to our knowledge there were no current or ongoing reviews on the topic of PCIC for older people.

## Methods

### Review design

A wide-ranging consideration of review types, including, scoping, systematic, rapid evidence, integrative, realist and narrative reviews, was undertaken. An integrative review was selected as the methodology for several reasons. Integrative reviews critique and synthesise current literature on a topic in an integrated way. They do this by synthesising a range of literature to provide a comprehensive understanding of a phenomenon, integrating the current state of knowledge to generate new insights [28,29]. Integrative reviews are suited to research contexts and phenomena involving multiple disciplines and fields [30]. This places integrative reviews as part of a ‘sensemaking/sensegiving circle of scholarship’ [31, p. 4]. Integrative reviews are unique in their capacity to provide broader summaries of literature, including findings from a range of research designs and diverse methodologies, to develop a holistic understanding of the topic, present the state of the science and contribute to theory development and evidence-based research, practice and policy development [32,33], which fit well the diverse nature of literature on integrated care [34,35]. Integrative reviews identify gaps in the literature and suggest future directions for research and practice [36]. Integrative reviews are useful for exploring healthcare phenomena [37,38] and used specifically in reviewing integrated health and social care [39] and care experiences [40]. Finally, integrative reviews are suited to the study of complex phenomena, such as integrated care, where a research question is clearly defined, as in the case of this review focusing on older people’s PCIC experiences [36,41,42].

Integrative reviews require a methodological approach which is transparent, rigorous, systematic and reproduceable [36]. Having reviewed existing guidance regarding the various steps which should be followed [36,43,44] the authors opted to follow the five stages advocated by Whittemore and Knafl (2005), namely: 1) problem identification, including definition of the research question and related objectives; 2) literature search, including developing search strategy and pilot literature search; 3) data evaluation with categorisation of included studies; 4) data analysis; and 5) presentation of the findings, including discussion of the results [42]. A protocol for this review was developed in advance.

### Literature search

The search strategy was developed, as per best practice, following expert advice from a qualified academic librarian [43] and designed to be as comprehensive as possible and aligned to the review question and objectives [42]. To address the research question of ‘What are older people’s experiences of PCIC?’, the PICo framework was applied. This framework considers P—population, context and/or problem situation; I—intervention; and Co—condition, and was applied as shown in Table 1. In deciding on search terms, the authors considered database subject headings and index terms, authors’ own knowledge of the subject and terms used in previous key studies. To retrieve the most relevant literature on older people’s experiences of PCIC, search terms included accepted synonyms for the main concepts of the search: older people; integrated care; person-centred care (PCC); and experiences of this care. Variations in terms and spelling across databases and regional and national contexts were considered. The terms ‘person centred/focused’, ‘patient centred/focused’ and ‘client-centred/focused’ were used due to their prevalence [45]. Subject headings and free text search terms were used and adapted for each database. There are current significant limitations in subject headings pertaining to PCC as they relate to the *delivery* of healthcare as opposed to the *experience* of it [46] as is also the case with subject headings relating to integrated care. Truncation was used, as recommended in integrative reviews [33,47].

**Table 1:** Search Strategy.


SEARCH STRATEGY	

**Population**	Subject headings: “Aged”; “Older Adulthood” Search terms: Elderly or “Older adult*” or “Older people” or “Older person*” or Aged or Geriatric* ) OR AB ( Elderly or “Older adult*” or “Older people” or “Older person*” or Aged or Geriatric*

**AND**	

**Intervention**	Subject headings: “Delivery of Health Care, Integrated; Integrated health care system; “Integrated Services”; “Continuity of Patient Care”; “Continuum of Care” Search terms: “Integrated care” or “Multi-disciplinary care” or “Multidisciplinary care” or “Inter-disciplinary care” or “Interdisciplinary care” or “Co-ordinated care” or “Coordinated care” or “Care coordination” or “Care co-ordination” or “Collaborative care” or “Integrated delivery” or “Integrated health care” or “Integrated healthcare” or “Inter-agency care” or “Interagency care” or “continuity of care” AND

	Subject headings: “Patient-Centered Care” Search terms: (“Person centred*” or “Person centered*” or “Person focused” or “Client centred*” or “Client centered*” or “Client focused” or “Patient centred*” or “Patient centered*” or “Patient focused”)

**AND**	

**Context**	patient* or client* or “service user*” or consumer*) N2 (experienc* or view* or perspective* or feel* or felt or prefer*) ) OR AB ( (patient* or client* or “service user*” or consumer*) N2 (experienc* or view* or perspective* or feel* or felt or prefer*

**AND**	

**Setting**	All healthcare settings

**FILTERS**	

**Language**	English

**Publication dates**	01/01/2014-30/07/2024

**Databases**	MedLine, CINAHL, PsycInfo, Embase, Web of Science.

**Date searches were run**	30 July 2024

**Field**	By title AND abstract.


The search strategy included five electronic databases: Medline, PsycInfo, CINAHL, Embase and Web of Science, three of which were accessed on EBSCO platform (Medline, PsycInfo, CINAHL); one on Ovid (Embase); and one on Clarivate (Web of Science). This selection followed expert advice from qualified academic librarians, as well as previous knowledge and experience of the review team. This selection was based on databases’ unique content, and intended to capture literature on healthcare, specifically PCIC, for older people, including medical and nursing sources, as well as studies applying sociological approaches to healthcare, social care and mental health. A pilot stage informed the finalised search strategy, including initial searches of all databases to identify publications on older people’s experiences of PCIC. The purpose of the pilot stage and related scoping searches was to gauge the volume of literature available and to test search terms. As there was not sufficient literature specifically using the term ‘person-centred integrated care’, integrated care AND patient centred care or person-centred care were combined to retrieve studies focusing on person-centredness in integrated care contexts, as outlined in Table 1. Various terms and date ranges were trialled and assessed for relevance. An outcome of this was that in addition to search terms relating to integrated care, PCC and older people, the concept of ‘patient experiences’ was added to increase the relevance of retrieved studies. Proximity operators were applied to retrieve experiences of the patient receiving care, as opposed to health care professionals (HCPs) and carer experiences.

The ten-year timeframe was selected as piloting showed that 2014 marked the emergence of the concept of PCIC [1]. This was further tested to ascertain whether, and if so, how many, publications would be missed by thus narrowing the timeframe. The date range 2004–2024 was tested and retrieved the same numbers of publications as 2014–2024, indicating the appropriateness of this date range.

Inclusion and exclusion eligibility criteria were applied, as detailed in Table 2. There were no restrictions on research methodology or design, care setting or geographical location.

**Table 2:** Summary of inclusion and exclusion criteria.


INCLUSION AND EXCLUSION CRITERIA FOR SELECTION OF PUBLICATIONS	

**Publication selection criteria**	Studies published between 1 January 2014–30 July 2024.

	Empirical studies on older people’s experiences of integrated care (older person defined as 65 years or older).

	Studies reporting on care experiences in any setting.

	Studies published in English.

**Exclusion criteria**	Studies published outside of the date range January 2014–30 July 2024.

	Studies not reporting on experiences of individuals aged 65 years and older.

	Non-empirical studies.

	Studies not explicitly referencing integrated care.

	Studies not published in English.


### Study selection, extraction and analysis

**Figure 1:**
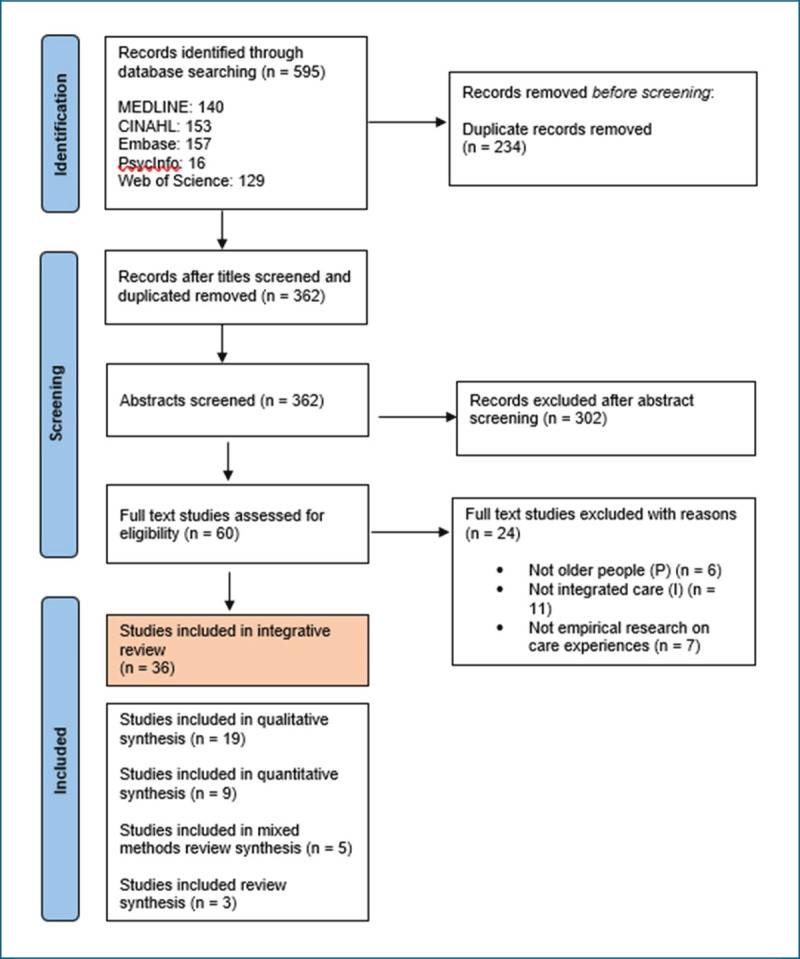
Preferred Reporting Items for Systematic Reviews and Meta-Analyse (PRISMA) flow diagram.

The selection of eligible studies for inclusion involved the following:

1. Following the pilot stage, the review team re-ran the finalised search strategies, screening all results of the electronic search. All records retrieved from searches were exported into EndNote [48] and deduplicated, followed by additional manual deduplication.
2. Screening against the inclusion criteria was conducted by three independent reviewers, using Rayyan (2024) software, which enabled blinded reviewing. Screening was conducted firstly on the publication’s title and abstract to determine whether or not a publication was eligible for potential inclusion.
3. Full texts of potential publications were sourced and screened to determine eligibility.
4. Reasons for exclusion of full text studies that did not meet the inclusion criteria were recorded and reported. Any disagreements that arose between the reviewers at each stage of the selection process were resolved through discussion.
5. Quality appraisal was conducted using appropriate tools. Tools were selected according to studies’ methodologies. The JBI tools for qualitative and quantitative research [49,50,51,52] and the Mixed Method Appraisal Tool (MMAT) [53] for mixed method studies were applied. In the absence of specific tools for scoping and meta-ethnographic reviews, the included reviews were subject to assessments consisting of questions concerning their methodologies, search strategies and analyses. Critical appraisal of all studies was conducted by the lead researcher with a selection additionally reviewed by the two other members of the review team.
6. The selection of studies was documented in a PRISMA flowchart: ‘Preferred Reporting Items for Systematic Reviews and Meta-Analyses’ [54], which outlined search and screening processes and outcomes, as detailed in Figure 1.

A common analytical framework was applied to ensure the same questions were asked and standardised information categories extracted from each study to ensure consistency in comparing and contrasting studies and to mitigate against bias [55]. Data extracted included author; date and country; study aim; setting and focus area; study design and data collection; sample; definition of integrated care used, if any; and findings regarding integrated care experiences. Extracted data was ‘charted’ in a standardised data extraction chart using Microsoft Excel.

## Results

A total of 595 studies were retrieved and thirty-six studies finally included.

### Quality Appraisal

All 36 studies included after full text screening were subject to critical quality appraisal. Overall, the quality of studies varied between satisfactory and high. None were excluded as even lower quality studies can illustrate empirical evidence [39,56]. Critical appraisal of all 36 studies was conducted by the lead researcher with a selection of assessments reviewed additionally by the two other members of the review team.

There were some specific features of the critical appraisal process. Only one of the nineteen qualitative studies [57] stated a philosophical perspective which problematised appraising congruity between the stated philosophical perspective and the research methodology, the first question of the checklist for qualitative research. Secondly, none of the 19 qualitative studies included a statement locating the researcher culturally or theoretically while most of the studies did include professional discipline-related information for the researchers as well as their specific training pertinent to qualitative research. The majority of studies referenced steps taken to mitigate subjective bias, as such addressing the influence of the researcher on the research, but none addressed the influence of the research on the researcher.

Of the nine quantitative studies, among randomised controlled trials (RCTs) (n = 2), the most common quality issues concerned differences between characteristics of participants in each arm; lack of concealment to participants and practitioners beyond baseline, due to delivery of face to face initiatives; assessment bias at outcome level, due to lack of concealment to researchers; delays in enrolment and programme initiation, potentially diluting impact; issues regarding co-location whereby control practitioners could have observed intervention practices; and inclusion of specific information regarding randomisation methods. Cross-sectional studies (n = 6) did not always include information regarding confounding factors. The quasi-experimental study (n = 1) also featured some differences across groups and lack of representation of the most frail older people. Regarding the mixed method studies (n = 5), some did not include adequate rationale for using a mixed methods design to address the research question. Finally, regarding the reviews, all (n = 3) were found to be high quality according to the assessment questions with respect to scoping and meta-ethnographic reviews as appropriate.

### Overview of included studies

**Figure 2:**
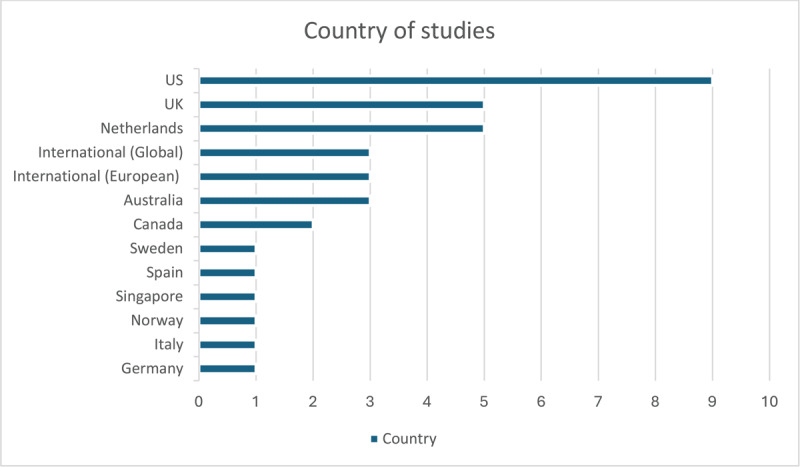
Country of studies.

Publication dates began in 2014 and went up to 2024, with half (n = 18) published between 2020–2022. Nineteen used qualitative methodologies, nine quantitative, five were mixed-method and three were reviews. The studies were based predominantly in the US and Europe and included over 15 countries worldwide and six international studies, as detailed in Figure 2.

**Figure 3:**
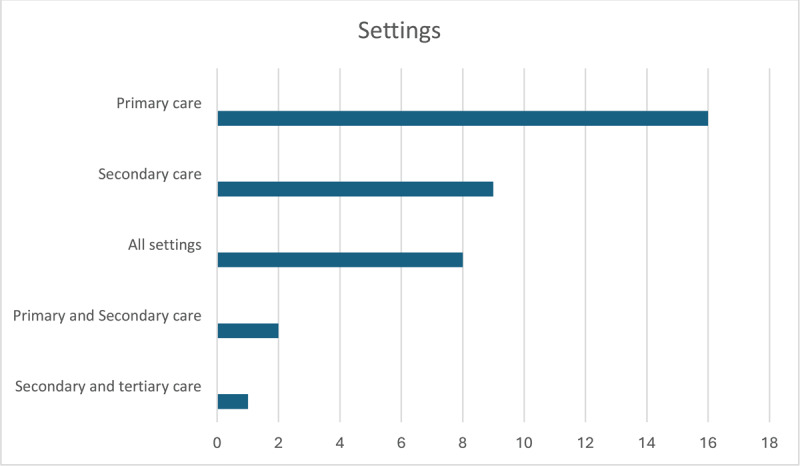
Settings.

Almost half (n = 16) of the 36 studies exclusively concerned primary care settings, as illustrated in Figure 3, with several noting the particular suitability of this setting for the provision of continuous coordinated care.

**Figure 4:**
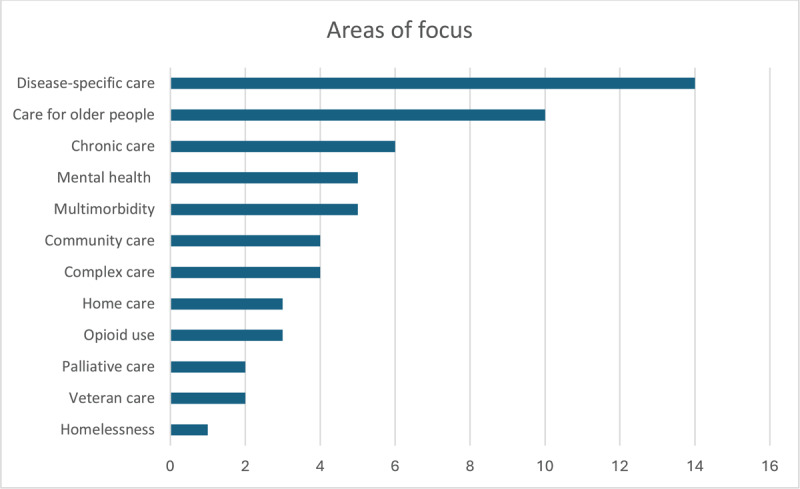
Areas of focus.

Studies focused on integrated care experiences within a wide range of areas, as shown in Figure 4. These included: care for older people (n = 10); chronic care (n = 6); multimorbidity (n = 5); mental health (n = 5); complex care (n = 4); community care (n = 4); home care (n = 3); palliative care (n = 2); veteran care (n = 2); disease-specific care: diabetes (n = 4), cancer (n = 2); respiratory (n = 1); HIV (n = 1); Rheumatic and musculoskeletal diseases (RMDs) (n = 1); heart disease (n = 2); neurological diseases (n = 2); and obesity (n = 1); opioid use (n = 3); pharmacy (n = 1); and homelessness (n = 1).

Nine of 36 (25%) studies concerned older people as the entirety of their samples, defined as over 65 years, with one study using 60 years and over [58]. The majority of studies (n = 27, 75%) included people under 65 years in addition to older people and others also included HCPs and carer participants.

### Themes

Thematic analysis was conducted, as used in integrative research and reviews [33,59]. Four themes were identified following thematic analysis of the data extracted from included studies and their findings related to older people’s experiences. This process was conducted independently by two members of the review team and themes finalised by consensus.

#### Definitions and components of integrated care

Half of all included studies (n = 18) included a definition or definitions for integrated care while the other half did not. Some articles included setting-specific understandings of integrated care. Articles also included definitions from the literature for integrated care [4,6,60,61,62,63], inter-professional patient-centred (IPPC) practice [64], person-centred coordinated care (PCCC) [65,66,67] and goal-oriented PCIC [20]. Ten of the studies mentioned the existence of multiple conceptualisations, values, terminology and models relating to and attributed to integrated care in literature and in practice. Integrated care was described as ‘conceptually ambiguous’ [68, p. 559] and PCIC as ‘awash with overlapping concepts and terminology’ [20, p. 2]. Studies noted that this multiplicity challenges measurement and problematises the development of evaluation frameworks and tools to assess outcomes of care. Fourteen studies asserted the lack of research on the perspectives and voice of the person (patient) relating to integrated care experiences. They asserted that frameworks for integrated care have been designed from professional provider, organisational or policy perspectives which do not encompass patient experiences or preferences related to relational aspects and outcomes of care [69]; or acknowledge that assessment of the quality of integrated care, and especially of PCIC, is best assessed by the patient [20]. Most evaluation of integrated care is quantitative, takes policy, organisation or service perspectives and uses tools designed by researchers or practitioners which do not include patient perspectives [70]. When measurement occurs, it is often confined to aspects of services rather than comprehensive assessment of integrated care experiences [71]. This lack of research on patient perspectives and experiences is especially true for older adults as was articulated in those nine studies exclusively focusing on this population.

**Figure 5:**
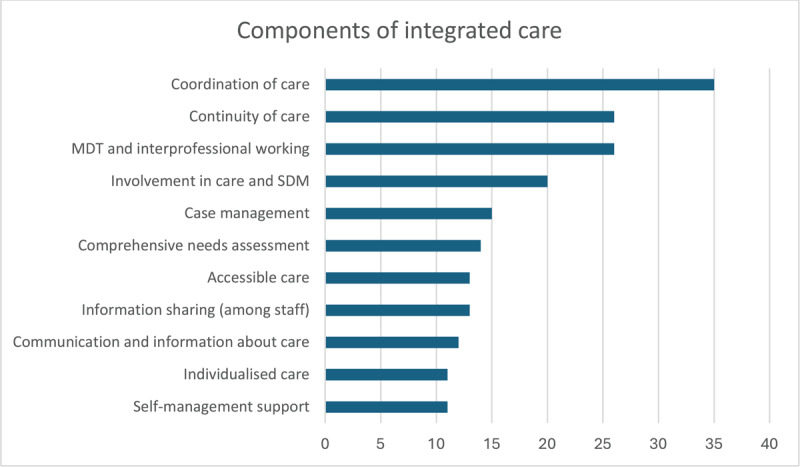
Components of integrated care.

All thirty-six studies outlined specific components of integrated care and PCIC or PCCC, as detailed in Figure 5. All but one study explicitly mentioned coordination of care, with the exceptional study [72] using synonyms for coordinating practices. Several components were prominently mentioned across included studies: continuity of care (n = 26); multidisciplinary team (MDT) and interprofessional working (n = 26); involvement of the person (patient) in care and shared decision-making (SDM) (n = 20), with some (n = 4) also mentioning the involvement of carers and family; case management models of care (n = 15); comprehensive needs assessment (n = 14); information sharing among HCPs and teams (n = 13); accessible care (n = 13); communication and information about care for the person (n = 12); self-management support (n = 11) and individualised care (n = 11). Components mentioned by a minority of studies included: individualised care planning including the person’s own goals (n = 5), links to community resources (n = 4), co-location (n = 2), shared practice tools, including guidelines and pathways (n = 2), pooled budgets (n = 2) and a collective attitude or ethos (n = 1).

#### Conceptualisations of person-centredness in the context of integrated care

Studies highlighted PCIC, as opposed to disease-specific and service-centred integrated care, as crucial for older people living with multiple and complex conditions to avoid fragmentation and cater for diverse and complex needs [73,74], leading to higher satisfaction with care and feelings of respect and equality [71,72,73,75]. PCC was considered a core element of integrated care [75,76,77,78,79]. Integrated care was also considered to enable PCC and the outcome of tailored care [80]. Some studies stated that a person-centred approach to integration was often more important to the patient than organisational aspects of care [81]. However, initiatives did not always achieve person-centredness from patients’ perspectives [77]. Studies mentioned multiplicity of definitions for PCC and the absence of a universal definition [77]. The following PCC components in integrated contexts were mentioned: relational care, therapeutic relationship and emotional support; providing tailored care informed by the person’s values and preferences and goals for care; person-centred communication; empowering and involving the patient in care and SDM; and holistic care that acknowledges needs of carers, families and wider ‘ecosystems’ of care [73,79,82,83,84]. Related HCP skills or competencies included interpersonal and communication skills [73].

Psychological and emotional distress caused by ageing, chronic illness and multimorbidity and the resulting impact on functionality, sense of self and social identity was often the most distressing for participants. The presence of trusted relational care and subsequent emotional and social support, sometimes in the form of a case manager, a single point of contact, was seen as a hugely important element of person-centredness [58,69,70,71,72,73,81,82,85,86,87,88]. Patients, including older people, repeatedly expressed their wish in included studies to be heard – ideally without having to repeat their stories to several specialists – seen and recognised as unique people with distinct care needs and wider lives [79,81,89,90]. This felt experience of being known contributed to feelings of safety and reassurance and alleviated those of overwhelm and isolation [71,79,81].

It follows that this wish becomes even more urgent in the context of multiple, changing – and often deteriorating – health and social circumstances. A majority of studies (n = 27) cited personalised holistic assessments of need, which incorporated the person’s values and preferences and involved them and their carers and families, and subsequent provision of tailored biopsychosocial supports as central to person-centredness in the context of integrated care. The flexibility of these supports organised around the person was found to be important in the context of changing needs [72]. In addition to involving people in assessment half of all studies (n = 18) cited the importance of empowering individuals receiving care through SDM, as well as involvement of their carers and families. Connected organisational and system issues included the extent to which HCPs were enabled to engage in relational care and have sufficient time for meaningful and respectful interactions as well as the involvement of patients in the evaluation of PCIC [73,81,83].

#### Integrated care as improving care experiences

While integrated care was found to improve care experiences when successfully delivered, experiences were mixed. Patients, including older people, were found to perceive and value integrated care and to appreciate and benefit from integrated practices which led to more efficient and responsive care [75,79,80,89,91,92,93,94]. Studies exclusively concerning older people reported better care experiences in integrated initiatives compared with non-integrated [74,94,95] and that older people valued receiving care in their communities and homes [77] and relational care [69,83]. Where studies included wider samples, including older people, better care experiences and higher satisfaction with care were reported for integrated than non-integrated initiatives [88]. Positive integrated care experiences featured benefits including coordinated care; continuous care, including relational care; personalised holistic care; effective communication and information about care; and empowerment and involvement in care. These benefits led to higher satisfaction with care, enhanced wellbeing and decreased depression, improved self-management, increased trust and improved and sustained relationships between patients and HCPs [96].

However, integrated care initiatives intended to improve care experiences often did not produce expected results. At times modest increases in satisfaction were reported in experiences with integrated care initiatives [86,97]. Some studies reported poor care experiences and dissatisfaction with care [75,80]. Low overall perceptions of integration and coordination of care were reported [78,80,84,89,93]. Other studies reported little difference or lack of impact on patients [57,95]. In some cases, higher satisfaction with integrated care was evident at six months but not at longer intervals [95]. Integrated care initiatives did not always reduce waiting times, hospitalisations and costs [57,75,76,86,98]. Care which was not integrated was perceived as more time-consuming and expensive for patients, often due to travel time and increased costs [98]. Women and marginalised groups had lower perceptions of person-centred coordination of care [84]; people with mental health issues lower experiences of coordinated care [99]; and women and people that were either older, unemployed, with comorbidities or lower self-reported health, or less engaged in their care had worse care experiences [80]. Patients living with severe illness and multiple conditions were found to suffer most from a lack of coordination [58].

#### Four components of PCIC evidenced as part of older people’s PCIC experiences

**Figure 6:**
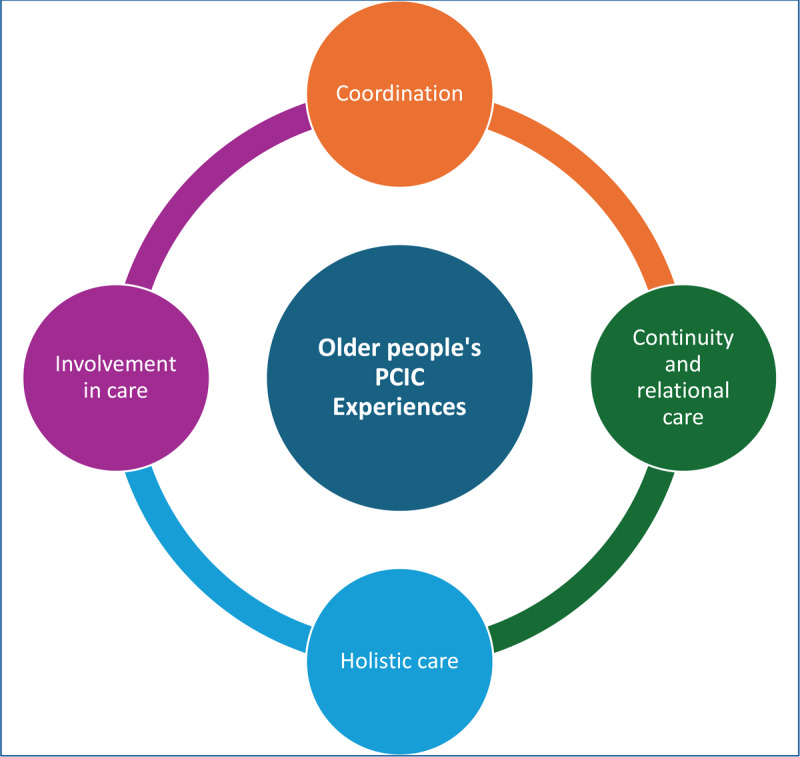
Older People’s PCIC Experiences.

Four components of PCIC were evidenced as part of older people’s PCIC experiences, as shown in Figure 6.

##### 1. Coordination

All thirty-six studies cited the importance of experiences of coordination of care as part of integrated care. Patients generally and older people particularly, articulated emotional distress and anxiety due to the burden of navigating fragmented health systems for their multiple and complex care needs [82,97]. Integrated care was perceived as alleviating this burden through improved coordination within MDTs and across HCPs and systems, including between physical and mental healthcare [58,70,73,83,87,91,93]. Some studies suggested that integrated care may be more crucial at certain times with increased risk of fragmentation, i.e. transitions and recurrence or decline in conditions. Where successful, integrated care was found to improve coordination of care, including seamless transitions across services and settings and information sharing and alignment across HCPs and teams, as well as with other health and social care systems, community resources and insurance providers [69,70,75,87,91,93]. This coordination was found to be hugely valued by older people [94]. This coordinated care led to timely and accessible (often local) multidisciplinary care and feelings of safety, security and reassurance that their health was being holistically and effectively managed [58,68,70,71,72,81,83,90,91,92,98].

Some studies reported coordination as existing at structural professional and systems levels but not experienced by patients and carers [57]. Patients, including older people, reported perceptions that HCPs involved in their care did not speak to each other and having to repeat their stories [58,90,93]. This led to negative experiences, lack of access to timely multidisciplinary care and feelings that HCPs did not understand their health issues or their values [75,76,90]. In some primary care interventions, this lack of coordination led to patients having to access emergency care services [75]. A lack of interprofessional communication, coordination and information sharing led to perceptions of inconsistent and fragmented care, resulting in negative care experiences and feelings of anxiety and feeling disrespected [76,90,94]. As mentioned above, people experiencing socioeconomic marginalisation or living with mental health conditions experienced poorer coordination and suffered more from insufficient coordination [58,84,99]. Gaps or challenges were also identified regarding identifying patients’ goals, developing and sharing individualised care plans with HCPs and with older people and monitoring progress [20,77,84] and assessment skills, especially regarding marginalised patients [84].

##### 2. Continuity and relational care

A majority of studies (n = 26, 72%) cited continuity of care, both management and relational continuity, as highly valued and reported that it facilitated coordinated care. Integrated care experiences were found to improve perceptions of continuity, including through case management models to assist in care coordination, regular comprehensive assessment and follow-up care. These case management models of care entailed trusted relationships with single points of contact to provide advocacy supports, anticipate changing needs and facilitate access to care [70,77,81]. In addition, integrated relational care for older people provided self-management support, positive communication and interactions, and continuous follow-up care, alleviated fears around ageing and ill-health, resulting in feelings of being known, improved resilience, personal growth and wellbeing and reduced depression, and relieved patients, carers and families from the burden of navigating and coordinating care [70,71,73,74,81,87,90,96]. Relational aspects of care and outcomes and having ample time to discuss concerns with trusted HCPs was especially important to patients, including older people [58,69,81]. Older people with multimorbidity were found to prefer liaising with a single provider to coordinate their care [73].

Discontinuity was experienced in relation to discharge practices [72] and transitions and follow-up care [76,85]. Long waiting times and a lack of accessible care also resulted in discontinuity and led to frustrations and poor care experiences [75,76,90,98]. Staff shortages and turnover impacted on experiences of discontinuity, a lack of access to appropriate care and a lack of follow-up care with patients [75,76,77,92]. Staff shortages and turnover and related discontinuity also resulted in a lack of time for relational care and meaningful interactions and assessments that were overly clinical and not sufficiently holistic from the perspective of older people [73,77,81,90]. Where relational care in the form of coordinator roles was provided reactively, rather than proactively to anticipate needs, this was reported as producing limited benefits for patients [83].

##### 3. Holistic care

Twenty-one of the thirty-six studies (58%) specifically cited integrated care as improving provision of holistic care and addressing social and emotional needs compared with non-integrated care. Integrated care was often perceived as closely connected to holistic care, providing a ‘one-stop shop’ for a spectrum of needs [57,69,72,94]. Some studies mentioned a consensus that integrated care for people with complex needs could not solely focus on physical health and had to adopt a holistic view of the person, including social needs [87]. Holistic care experiences included personalised care that incorporated the person’s values, preferences and life goals [20,87,90]. There were expectations on the part of patients that long-term goals would be addressed as part of care planning [20]. Holistic care also provided social care and practical supports, often relating to cleaning and maintenance work in the home [68,90,91]. Studies mentioned the importance of care being linked with community resources in order to ensure sustainable social and other supports [71,89,90,93]. Some studies cited the need for assessment and care processes to be more holistic and personalised to encompass emotional and psychological supports and the needs of carers and families [57,76,81,90].

##### 4. Involvement in care

Almost two-thirds of the studies (n = 23, 63%) cited the importance of the involvement of the person in integrated care, through effective information and communication about care and more focused practices such as SDM and partnership in care. Involvement in care was mentioned as especially important in the context of fears around ageing and chronic illness. Fears were repeatedly expressed in the studies regarding losing functionality, independence, social connection and ultimately control over their lives [71,73,74,81,83,90]. Positive communication and information about care and related interactions were seen as essential to positive integrated care experiences [58,71,72,73,74,76,79,82,92]. Older people were specifically evidenced to value and expect interprofessional communication about their care [94]; comprehensive communication and information about their care from all providers [58]; clarity regarding what they can and cannot expect from integrated care experiences [74,77,81,100]; and who the, often many, members of integrated care teams were to ensure understanding, trust and comprehensive needs assessment [77].

Studies reported patients and older people’s dissatisfaction when insufficient time was afforded to consultations [72,73,81]. Older people reported finding it difficult to voice dissatisfaction with care to professionals involved, sometimes due to a lack of understanding of their rights and entitlements or cultural or cognitive issues [77]. At times, deficits in communication and information were evident as well as lack of understanding among patients of the concept of integrated care, leading to a lack of trust in HCPs and staff communication training was called for in this area [74,76,81,83,84,97]. People with mental health issues were found to experience poorer communication and increased knowledge fragmentation [99]. Older people not sufficiently understanding integrated care interventions and what they could expect from them limited their capacity to engage with and benefit from them [74,77,100].

Integrated care models engaged and involved people in SDM to ‘grow’ care plans collaboratively and empower and enable patients to participate in their care [72,79,87]. Some studies cited integrated care experiences as increasing involvement of patients, carers and families [74]. Integrated models also provided self-management support to assist individuals in coping with ageing and health conditions and related social issues [58,68,70,71,92,97]. Some patients reported and valued feeling involved [68]. Others resented not being more involved [94]. The majority of MDTs did not include older people as members [77]. There were also varying preferences found among patients in general and older people regarding participation in care planning and discussions about care. Many hugely valued this involvement, including people living with long-term degenerative conditions [68]. Others did not wish to be as active in this area, and this was often impacted by health status and trajectory [79,94]. Different opinions were also expressed in terms of information sharing about their health across HCPs and teams, with some older people expressing concerns about privacy and wishing to give specific consent as appropriate [93]. The need to further involve families and carers was mentioned also [76,90].

## Discussion

This review adds to the limited but growing body of knowledge regarding older people’s experiences of integrated care. Firstly, this review confirms that PCIC experiences are often conceptualised using multiple and varied definitions and attributes of integrated care. The multiple components cited as constituting integrated care aligns with existing literature in this area and is testament to the multiplicity of values, components and activities which are attributed to integrated care [6]. The identified components also align with guidance on integrated care for older people [101]. The fact that half of included studies did not include a definition indicates a lack of focus on conceptualisation in empirical research on integrated care for older people.

Secondly, this review illustrates the symbiotic relationship between PCC and integrated care and the aspects of person-centredness ideally present in integrated care contexts. This resonates with assertions that person-centredness is key for quality integrated service user care experiences and that integrated care is a core component of PCC [19,102]. This also aligns with WHO recommendations for care for older people with multimorbidity to be person-centred, holistic and integrated [103,104]. A PCIC approach is especially suited to and necessary for older people living with multiple complex conditions and requiring continuous care for dynamic needs who are at high risk of experiencing fragmentation [60,105]. Coordination of this care serves to address fragmentation that they would likely otherwise experience in accessing care from disparate specialist teams. Person-centred values evidenced include relational care, incorporating the person’s values and preferences for care, as well as related, and often significantly more important to the person, life goals into care planning and delivery, and involving them in decisions about this care in the context of changing needs [106,107].

**Figure 7:**
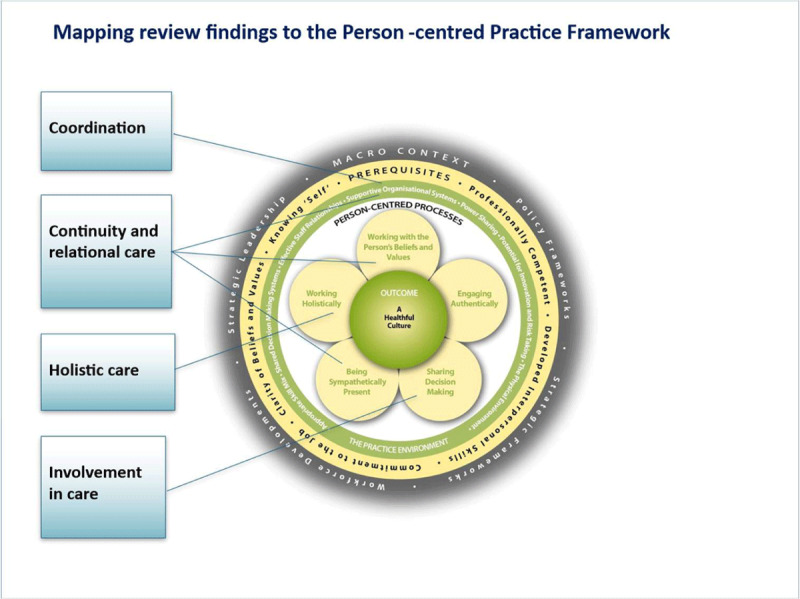
Mapping review findings to the PCPF. Source: McCance and McCormack 2021.

Findings also resonated with the Person-centred Practice Framework (PCPF) [24] as illustrated in Figure 7. The PCPF centralises the establishment of the therapeutic relationship between staff and the patient, including their families and carers and identifies person-centred processes, including provision of tailored holistic care, which is informed by the person’s beliefs and values, involving them as a partner in care through SDM while engaging authentically with the person and being sympathetically present. These processes ideally lead to the outcome of a positive care experience where the person feels a sense of wellbeing, involved and respected within a healthful culture of care. Figure 7 connects the components of PCIC identified in this review with the relevant PCPF process and practice environment domains. Coordination resonates with the practice environment; continuity and relational care with both the practice environment and with processes of working with person’s beliefs and values and being sympathetically present; holistic care with working holistically, and involvement in care with SDM.

Thirdly, this review has found that integrated care can optimise care experiences when implemented successfully and that older people’s experiences of PCIC are varied. Positive PCIC experiences for older people included: experiences of coordination and continuity of care, relational care, being involved in care, effective communication and information about care and personalised holistic care. These experiences are highly inter-related. Relational care, for instance, can facilitate experiences of continuity, coordination, effective communication, involvement in care and enable knowing the person, leading to enhanced holistic care. These findings regarding positive PCIC experiences resonate with literature on integrated care and PCIC for older people. This literature asserts that: experiences of coordination [105] and of management or team and relational continuity lead to higher care satisfaction among older people [108,109]; relational care, including case management models, enhance care experiences and are hugely valued [110,111]; involvement in care, including effective communication and information about care, SDM and collaborative practices, is highly valued [1,112,113,114]; and person-centred holistic care assessment and delivery is central to PCIC approaches [106,115].

Empowerment is often – ideally – at the heart of integrated care, which seeks to shift power, recognise the expertise of the person and centralise their experience and wider outcomes for their lives [3]. Older people have been found to significantly value this empowerment or involvement in care, often manifest in integrated care acknowledging patients’ active personhood, unique preferences and capacity to engage in care [7]. This review’s findings also resonate with literature on integrated care and PCIC which emphasises the importance of being seen, heard and respected [1,116], person-centred approaches for older people with multimorbidity [117] and related enabling person-centred HCP skills and attributes [1,117].

This review also confirmed the existence of particular challenges in successfully implementing PCIC, aligning with literature extolling the need for supportive PCIC organisational contexts, which may require practical and conceptual changes [1,2,115]. Studies further confirmed a stark knowledge and evidence gap regarding experiences of integrated care in general [27] and especially older people’s experiences [26]. Older people perceive and value integrated care and benefit from coordination that is continuous, person-centred, tailored to their holistic needs and enabling their involvement if they so wish. However, this review has confirmed that progress has been inconsistent, and implementation beset by challenges. Experiences were mixed and implementation of PCIC approaches plagued by structural and systemic issues at micro, meso and macro levels. These included the need for HCPs to be afforded sufficient time to facilitate meaningful interactions to get to know patients, and the lack of coordination within and across healthcare services and systems. The need for further coordination was identified between health and social care infrastructure and policies on the one hand and wider community resources and actors on the other. These actors play a significant role in addressing socioeconomic determinants of health and wellbeing to better support older people living with multiple chronic and complex conditions.

These findings resonate with recent critiques of integrated care and PCIC. Critiques concern the lack of value placed on relational and relationship-oriented care as a priority by organisational management and system design, at odds with the considerable value placed on it by patients, often far above other (task-based) aspects of care [7]. Critiques further identify the need to meaningfully involve the person, their families and communities more in care delivery and to have corresponding justifying institutional logics at all levels of care; to effectively link with informal and community care supports, and to provide more support for HCPs around competencies and capabilities [16,115,118,119].

Fourthly, evaluation of PCIC is complicated by the aforementioned multiplicity of definitions, conceptualisations and models of care. The complexity of these models makes proving outcomes challenging. Many of the studies’ methodologies sought to bring consistency around components to achieve more systematic evaluation. It is clear from analysis of the included studies that evidence on older people’s experiences in the qualitative studies privileges the voice of the older person more and as such may be more appropriate to capturing PCIC experiences, albeit in conjunction with other research designs. These studies include perspectives in the older person’s own words and bring an enriched sense of their feelings, preferences and values regarding care. Many integrated care frameworks and models were developed from policy, organisational and service perspectives, rather than patient or service user perspectives. Evaluation of integrated care is made difficult by its myriad forms, conceptualisations, terminology and models. This review reiterates claims that where evaluation has taken place, it has largely been to evaluate systemic, organisational or clinical processes within care rather than the patient’s experience [1,26]. Studies referenced that the scarce tools that do exist have been designed by professional ‘experts’ without sufficient input from the patient and as such risk missing opportunities to measure aspects of this care that are important to them, notably relational care [20,69,70,71]. This is especially true of older people. It is therefore vital that patients are involved in the evaluation of PCIC approaches [1,120].

## Strengths and limitations of this review

The strengths of this review include its focus exclusively on older people’s experiences through a rigorous and transparent integrative review methodology capable of i) capturing the complexity of the phenomenon of older people’s experiences of PCIC across settings and disciplines [32,42] and ii) analysing diverse research literature using multiple designs, evaluating the quality of the evidence and identifying knowledge gaps [33,121]. This review has been limited by documented challenges inherent in this methodology, including the complexity of incorporating diverse methodologies and implications on generalisability, potential selection rigour or bias and lack of clear guidelines [36,42]. Given the limited publications available, studies were included from a range of healthcare contexts and settings, involving older people with varying health and social circumstances, which impacted on potential specificity. Studies were included which included older people as part but not the entirety of samples. Given the scarcity of available research, studies were included which, while appraised by relevant tools, had weaker methodologies which may have limited their insights. Specificity has also likely been impacted by included studies’ use of varying definitions and terminology for integrated care. This review did not include grey literature or studies in languages other than English and was limited to the specified publication date range. Studies published since the literature search was performed in 2024 are thus not included in this review. These risks were mitigated by the review team being comprised of researchers experienced in clinical practice and research. Study screening and selection processes were performed independently by three researchers. Where findings concerned multiple groups, only findings pertaining to older people’s experiences were included. All members of the review team were cognisant of maintaining high standards of ethics and methodological rigour to ensure transparency.

## Conclusion and implications

This review has synthesised evidence on older people’s experiences of PCIC from empirical studies across medical, nursing, mental health, psychological, sociological and systems research fields. This review has confirmed the lack of research on older people’s experiences of PCIC and that older people, often living with multiple, complex and chronic conditions, are ideally placed to benefit from PCIC to mitigate against fragmentation they are otherwise at high risk of. When successfully implemented, PCIC resulted in benefits, including experiences of coordination and continuity of care; relational care; being involved in care, including effective communication and information about care; and provision of personalised holistic care.

Despite these benefits of PCIC for this population amid enhanced risks of care fragmentation, progress is indeed slow, often due to challenges in implementation and varying definitions and understandings, and results mixed regarding care experiences. Specific challenges or barriers were evident in included studies regarding unclear and inconsistent communication and information about care being provided to older people, preventing understanding of integrated care and related expectations of it. Systemic issues relating to the integration and organisation of care, lack of communication and coordination among HCPs and teams, and staff shortages prevented timely access to care and led to a lack of relational and management continuity. These issues meant that integrated care often did not reduce hospitalisations or costs and at times remained an organisational phenomenon which had very little discernible impact on the person’s care experience. Meaningful integration is challenged both by professional silos in provision of healthcare and also by a distinct lack of linkages with social care and community resources, central to holistically supporting people in their communities as they age.

This review’s findings have implications for further research on PCIC experiences of older people. Its findings, concerning components of PCIC evidenced as part of older people’s PCIC experiences, inform future research on the person-centred outcomes of integrated care for service users. These findings also contribute to conceptual understandings of PCIC as it manifests in care for older people. There is significant scope to further include in research the voice and perspectives of people with lived experience in general, and older people, in particular. This would strengthen future research on PCIC experiences and enable the co-design of evaluative tools and practices to assess these experiences in meaningful, person-centred ways.

## Additional File

The additional file for this article can be found as follows:

10.5334/ijic.9066.s1 Supplementary file 1. Summary of included studies.

## Author information

Sarah Murphy is a PhD Researcher at the Atlantic Technological University, Ireland. Sarah was awarded a Connaught Ulster Alliance Bursary for her PhD Study on service user experiences of person-centred integrated care in the context of the National Integrated Care Programme for Older Persons (NICPOP). Sarah has worked in research and evaluation in the fields of social policy and public health for over 20 years. She has particular interests in the social policy of health and social care, service user experiences of care in integrated contexts, and person-centredness in care environments.
