# Supplementary material for: How Do Older People Experience Person-Centred Integrated Care? An Integrative Review of the Evidence
Source: Int J Integr Care. 2025 Dec 15;25(4):21. doi: 10.5334/ijic.9066 (PMC12716249; doi:10.5334/ijic.9066)
Supplement: Supplementary file 1. — Summary of included studies. [file ijic-25-4-9066-s1.pdf]

Supplementary File 1: Summary of included studies

Table 1: Summary of included studies

Author, date and country	Study aim/ purpose	Setting and focus area	Study design and data collection	Sample	Definition of IC used	Findings regarding IC experiences
Banfield et al, 2017 [69]; Australia	To understand consumer and provider experiences of integrated primary health care	Primary care; Communit y care	Qualitative: Interviews.	19 patients with chronic conditions, 10 HCPs.	The terms integration and integrated care describe the complex, patient-centred strategies to improve coordination of healthcare services; 'integration' refers to structures and processes, while 'integrated care' is more concerned with the patient experiences and outcomes of such processes [4].	Consumers' experience of integrated care tended to be implicit in their descriptions of primary healthcare experiences more broadly. Experiences related to the typologies involved clinical and functional integration, such as continuity of providers and the usefulness of shared information.
Benzer et al, 2019 [99]; US	To determine how cardiovascular and mental health comorbidities relate to patient-centred coordinated care in the Department of Veterans Affairs.	Primary care; Diabetes; cardiovasc ular; mental Health; multimorb idity, veteran care	Quantitative : Cross-sectional survey	5806 patients with diabetes with Cardiovascula r and Mental Health Comorbidities .	Integrated care is a framework for representing care coordination from a patient-centred perspective.	Mental health conditions were associated with significantly lower patient experiences of coordinated care.
Berntsen	To explore, apply, refine and	All care	Qualitative:	19 people	Goal-oriented PC-IC: where the	Participants consistently reviewed care quality by how care

et al, 2018 [20]; Norway	operationalize quality of a PCIC framework, based on patients' evaluation of care	settings; Chronic care.	Interviews.	with long-term complex care needs.	person's overarching goals drive decisions about care.	supported/ threatened their long-term goals. Participants expected care to address their long-term goals and placed responsibility for care quality and delivery at the system level. The PC-IC process framework exposed system failure in identifying long-term goals, provision of shared long-term multimorbidity care plans, monitoring of care delivery and goal evaluation. This first version of a PC-IC process framework needs further validation in other settings
Bilodeau et al, 2015 [85]; Canada	To present results of a case study documenting IPPC practice carried on by teams within the oncology care continuum.	Secondary; Cancer care	Qualitative: Observations and interviews.	11 patients and loved ones.	PCNF definition of IPPC: the team works with the patient's beliefs and values, is involved, demonstrates empathy, shares decision making with the patient and provides holistic care [64].	Results suggest that IPPC practice is variable within teams, but optimal at the beginning of treatments or cancer recurrence. However, patients can experience breakdowns in the continuity of care and more difficult transitions between oncology continuum periods (diagnosis, treatment, follow-up).
Burridge et al, 2017 [72]; Australia	To explore patients' views of a new model of integrated care for patients with type 2 diabetes	Primary care; Diabetes care	Qualitative: Interviews.	30 patients living with diabetes.	None provided	Patients valued care organisation, i.e. structural elements of the clinic. For most patients, it was an enabling and motivating experience which included convenience, flexibility and prompt communication back to the referring GPs. Collaborative patient–clinician relationships played a key role in patients' engagement with advice and self-care. Most participants valued this model of care to manage variable and complex needs.
Coventry et al, 2015 [96]; UK	To test the effectiveness of an integrated collaborative care model for people with depression and long-term physical conditions	Primary care; Mental health, chronic care, multimorbidity.	Quantitative: RCT.	387 people with history of diabetes, heart disease, or both, who had depressive symptoms.	None provided	The mean depressive scores were lower in the collaborative care intervention arm. Patients in this arm rated their care as more patient-centred, were more satisfied with their care and reported improved self-management.
Cruz et al, 2021 [83]; UK	To design and evaluate person-centred integrated palliative and end-of-life care	Secondary care; Palliative and end of life care	Mixed method: Case study using records and	Total: 2 participants: 75-year-old patient with progressive supranuclear	None provided	There were 35 care providers from the public, private and the third sector, demonstrating that only the patient or carer can identify the ecosystem of care. The majority of care was for respite and the carer provided an average of four hours of care per every respite care hour. The method was replicated successfully.

			interviews.	palsy and (spouse) carer.		
Czypionka et al, 2020 [87]; International (Europe)	To identify success factors and crucial elements in the process of integrated care delivery for persons with complex needs from the practical perspective of the involved stakeholders	Primary and secondary; Complex care	Qualitative: Interviews and document analysis.	233 participants: 28 patients, 100 professionals, 19 caregivers, 86 managers and others.	Integrated care is defined as structured efforts to provide coordinated, pro-active, person-centred, multidisciplinary care by two or more well-communicating and collaborating care providers either within or across sectors [63].	Four categories that emerged from the overarching analysis are: (1) a holistic view of the patient, considering both mental health and the social situation in addition to physical health, (2) continuity of care in the form of single contact points, alignment of services and good relationships between patients and professionals, (3) relationships between professionals built on trust and facilitated by continuous communication, and (4) patient involvement in goal-setting and decision-making, allowing patients to adapt to reorganised service delivery.
Davis et al, 2018 [70]; US	To examine patient experiences of care in integrated behavioural health and primary care settings	Primary care; Community care, behavioural health care, mental health	Qualitative: Interviews and a MDT analysed data using a qualitative approach	24 patients.	Integrated care is a patient-centred approach designed to identify and address the majority of a person's physical and behavioural healthcare needs irrespective of setting [70].	Nineteen patients described receiving integrated care. Both primary care and CMHC patients: i) felt cared for when the full spectrum of their needs, including physical, emotional, and social circumstances, were addressed; and ii) perceived similar personal, interpersonal, and organisational benefits from integrated care, including personal growth, improved care quality, and access to care. Interactions with integrated team members helped patients develop and/or improve coping skills; patients shared lessons learned with family and friends. Service proximity, provider continuity and trust, and free initial behavioural health appointments supported patient access to, and engagement with, integrated care. In contrast, patients' prior experience, provider "mismatch," clinician turnover, and restrictive insurance coverage presented barriers in accessing and sustaining care.
Ericsson et al, 2021 [81]; Sweden	To obtain a better understanding, from the patients' perspective, the experience of CGA-based care by a geriatric mobile geriatric team (GerMoT).	Primary and secondary care; Community care, care for older people	Qualitative: Interviews.	22 older people, 11 relatives of older people.	None provided	The main finding expressed by the participants and their relatives was their feelings related to safety and security and being recognised. Participants found the care easily accessible and delivered according to needs by HCPs who knew them. Participants valued recurrent health examinations occurring and being given the time needed when seeking health care. Some participants found the information about the intervention to be unclear especially regarding which HCPs to contact.

Gabrielian et, 2021 [88]; US	To assess if primary care teams and integrated care tailored for homeless patients provide this population with superior experiences than mainstream primary care.	Primary care; Complex care, homeless ness	Quantitative: Cross-sectional survey.	1095 people experiencing homelessness with serious mental illness, 52 clinicians.	None provided	H-PACT respondents were more likely than mainstream providers to report favourable experiences and less likely to report unfavourable experiences in all 4 domains. Of 29 H-PACTs, 27.6% had high integration. High integration H-PACT respondents were twice as likely as low integration H-PACT respondents to report favourable access/ coordination experiences.
Ginting et al, 2022 [74]; Singapore	To understand patient experience of an integrated Patient Centred Medical Home (PCMH) model for complex community-dwelling older adults in Singapore.	Secondary care; Community care, care for older people	Mixed methods: Survey and focus groups	184 patients receiving home care.	None provided	Results suggest better care experience in PCMH than usual care. There were improvements in the CG-CAHPS measures on patient–provider communication, care coordination, office staff interactions, support for patients in caring for their own health, and provider rating in PCMH relative to usual care. Participants reported benefits of consolidated appointments and positive experience in sustained patient–provider relationship, SDM and family/caregiver engagement in PCMH. Participants may not fully comprehend the concept of integrated care, hindering both the effective communication of the intended care model and perceived benefits such as the provision of MDT-based care.
Grub et al, 2019 [82]; US	To explore experiences with opioid-related care under aggressive tapering efforts, monitoring and oversight among patients with chronic pain in an integrated delivery system.	All care settings; Opioid use, chronic pain	Qualitative: Interviews.	97 patients with chronic pain.	None provided	Many patients taking opioids experience debilitating physical side effects; navigating opioid treatment contributes to significant emotional distress among many patients with chronic pain; and the quality of patients' relationship with their primary care provider can be negatively affected by negotiations regarding long-term opioid treatment for chronic pain.
Hoedemakers et al, 2022; [95] Netherlands	To evaluate the value of the person-centred, integrated care programme Care Chain Frail Elderly (CCFE) compared with usual care.	Primary care; Care for older people, home care	Quantitative: Quasi-experimental, multicriteria decision analysis (MCDA)	384 community-dwelling older people living with frailty.	None provided	At 6 months, the overall value scores of CCFE were higher in all stakeholder groups, including patients, driven by enjoyment of life and person-centredness. At 12 months, the overall value scores in both groups were similar from patients' perspectives.
Hughes et	To explore how and why	All care	Qualitative:	20 patients.	Integrated care is an aim and a	Integrated care, intended to help patients manage their long-term conditions and avoid hospital admission, was only a small

al, 2022 [57]; UK	efforts to integrate health and social care failed to produce desired outcomes.	settings; Integrated health and social care	ethnograph y, in-depth case study		method for organising health and care services, particularly for older people and those with chronic conditions. It can be defined as an organising principle for health and care delivery and a set of initiatives and service models aimed at realising person-centred coordinated care (PCCC) in the face of population challenges such as ageing and increasing multi-morbidity [67].	part of the complex network that sustained patients at home. The structures of integrated care were of limited relevance and availability to patients' daily lives and were unable to compensate for changes in patients' health. As such patients' experiences remained largely unaffected and hospital admissions were not easily avoided.
Kanat et al, 2021 [73]; Germany	To assess the perspective that older patients with multimorbidity have of patient-centered care and to examine the transferability of the integrative model of PC to this population.	Secondary care; Multimor bidity, care for older people	Qualitative: Focus groups	20 older people with multimorbidit y.	None provided	All dimensions of the integrative PC model were confirmed for older patients with multimorbidity, including eight dimensions (individual care needs related to aging and chronic disease, biopsychosocial perspective, clinician-patient communication, essential characteristics of the clinician, clinician-patient-relationship, involvement of family and friends, coordination and continuity of care, access to care) were complemented by aspects specific to this target population.
Knowles et al, 2015 [91]; UK	To assess whether collaborative care that integrates depression care within the management of long-term conditions is implementable in UK primary care, and acceptable to patients and HCPs.	Primary care; Mental health, diabetes, chronic care, coronary heart disease	Qualitative: Interviews	31 patients with depression and diabetes, 30 HCPs.	None provided	Patients and professionals valued integration and collaborative ways of working because it enhanced co-ordination of mental and physical health care and provided a sense that patients' health was being more holistically managed. Patients and professionals articulated a preference for therapeutic and spatial separation between mental and physical health. Patients especially valued a separate space outside of their LTC clinic to discuss their emotional health problems.
Lawless et al, 2020 [58]; Internatio	To systematically map and synthesise the literature on older adults' perceptions and experiences of integrated	All care settings; Care for older	Scoping review	Review concerns studies with older people,	Integrated care is an approach to strengthen people-centred health systems through the promotion of the comprehensive delivery of	Patients expressed a desire for continuity, both in terms of care relationships and management, seamless transitions between care services and/or settings, and coordinated care that delivers quick access, effective treatment, self-care support, respect for patient preferences, and involves carers and

nal (global)	care.	people		defined as adults aged 60 years and above.	quality services across the life-course, designed according to the multidimensional needs of the population and the individual and delivered by a coordinated multidisciplinary team of providers working across settings and levels of care [62].	families.
McDonald et al, 2023 [79]; Australia	To explore patient, caregiver and GP perspectives of an integrated respiratory and palliative care service.	Secondary and tertiary care; Palliative care, respiratory care	Qualitative: Interviews	10 patients, 8 caregivers, five GPs.	Integrated care is the provision of disease-orientated care along with palliative care.	Patients valued integrated care, i.e. provision of disease-orientated care along with palliative care. Patients also valued communication, engagement and collaborative planning between patient, caregiver and HCPs and the delivery of PCC where HCPs listen 'and you are not treated like a number'. Various preferences were expressed regarding collaborative planning, while some patients found it helped, others described being too ill to participate. There were divergent preferences regarding engagement in discussions about future care.
Ngangue et al, 2020 [92]; Canada	To explore the perceptions and experiences of HCPs, patients and caregivers with a patient-centred interdisciplinary pragmatic intervention for multimorbidity in primary care.	Primary care; Multimorbidity	Qualitative: Interviews	30 participants: patients (9), caregivers (5), HCPs (16).	None provided	The programme allowed patients to adopt realistic and adapted objectives; to customize interventions to the patient's reality; and to help patients gain confidence, improve their knowledge, skills and motivation to manage their condition. Interprofessional collaboration eased the exchange of information via team meetings and electronic medical records. Challenges related to collaboration, communication, coordination of work and integration of newly relocated HCPs mainly due to part-time assignments and staff turnover. HCPs' part-time schedules limited their availability and hindered patients' follow-up.
Parra-Vega et al, 2022 [78]; Spain	To develop and validate a questionnaire for measuring patients' perception of integration across health care teams and social services.	All care settings; Interprofessional care	Quantitative: Cross-sectional survey	279 patients.	Integrated care is 'patient care that is coordinated across professionals, facilities, and support systems; continuous over time and between visits; tailored to the patients' needs and preferences; and based on shared responsibility between patient	The model explained 51% of the variation in the data and Cronbach's alpha was 0.8. Two factors comprising perception of coordination and assessment of patient-centred care were identified. The overall perception for integration was low. The reliability and validation of our questionnaire showed its potential as a valuable instrument for assessing patients' perception of the integration of care

					and caregivers for optimizing health' [60, p.113].	
Reynolds et al, 2021 [100]; International (Europe)	To explore experiences of integrated care from the perspective of service users.	Secondary care; Care for older people.	Mixed methods: Survey and qualitative consultation	228 older people.	The concept of PCCC/ P3C explicitly recognises the multidimensional nature of care experiences. P3C places an emphasis on understanding the relationship between individuals and their capabilities and resources, also acknowledging that care and support should strive to be responsive and coordinated across sectors, irrespective of organisational structures and configurations [65].	Results indicate the relevance of face-to-face administration for obtaining such an amount of data in this population group. The presence of a carer increased inclusion of more vulnerable respondents, such as the cognitively impaired, but posed a challenge in data interpretation. Open questions can lead to diverging and sometimes narrow interpretations by respondents. Cognitive impairment and a higher educational attainment were associated with lower levels of perceived person-centredness of care.
Rijken et al, 2021 [93]; Netherlands	To report on the patient perspective and explore the priorities, underlying values and preferences for care of people with multimorbidity.	Primary care; Chronic care, multimorbidity	Mixed method: questionnaire, focus groups, interviews.	863 people with multimorbidity.	None provided.	Frequently prioritized elements of care were the use of shared electronic health records, regular comprehensive assessments, self-management support and SDM, and care coordination. Preferences for how these elements should be specifically addressed differed according to individual values (e.g. weighing safety against privacy) and needs (e.g. ways of coping with multimorbidity).
Rijken et al, 2022 [84]; Netherlands	To assess the internal and construct validity of the Dutch P3CEQ to capture the experience of PCCC of people with chronic conditions.	Primary care; Chronic care	Quantitative: Cross-sectional survey	1098 adults with chronic conditions.	Person-centred coordinated care: 'care and support that is guided by and organized effectively around the needs and preferences of individuals', and is a comprehensive approach to care incorporating changes into organizational structures and behaviours of care professionals and service users [66].	The two-component structure found was similar to the UK validation study and sociodemographic correlates also resembled those found in the UK. Women, persons who were less educated, less health-literate or less activated experienced less person-centred coordinated care. P3CEQ scores correlated positively with GP performance scores and quality ratings of the total care received.

Schneider et al, 2023 [97]; US	To describe patient experiences with a phone-based and pharmacy-led opioid tapering program within an integrated health care system.	Primary care; Opioid use, pharmacy	Qualitative: interviews	25 participants in pharmacy-led opioid tapering program.	None provided	Most (60%) described a positive and satisfying experience with the tapering programme. Strengths reported by patients included a patient-centred and compassionate approach, flexible pace, accessible and knowledgeable pharmacist advocates, and improvements in quality of life (e.g. increased energy). Challenges reported included unhelpful or difficult-to-access nonpharmacological pain management options, negative quality of life impacts (e.g., inability to exercise), and lack of choice in the taper process. Following the programme, most patients (72%) described pain as reduced or manageable and expressed willingness to reuse the programme.
Singh et al, 2021 [98]; International, global	To review patient perspectives on integration of healthcare for HIV, type 2 diabetes and hypertension.	All care settings; HIV, hypertension, diabetes	Scoping review	Patients with HIV, hypertension and type 2 diabetes.	Integrated care is 'an approach to strengthen people-centred health systems (…) delivered by a coordinated multidisciplinary team of providers working across settings and levels of care' [62].	Patient's experiences with integrated care were reduced HIV-related stigma, reduced travel and treatment costs and a more holistic PCC. Prominent concerns were long waiting times at clinics and a lack of continuity of care in some clinics due to a lack of HCPs. Non-integrated care was perceived as time-consuming and more expensive.
Spezia et al, 2023 [80]; Italy	To provide an initial overview of the patient experience of integrated care from the perspective of people living with RMDs.	All care settings; RMDs.	Quantitative: Cross-sectional survey	433 people with RMDs.	Based on multidisciplinary working and the continuity and coordination of different levels of care, integrated care aims to go beyond the fragmentation and isolation of health services. In an integrated care setting, these services are designed and delivered in response to chronic and continuous patient health needs besides episodic ones [6].	Two factors (namely, "PCC" and "Health service delivery") were extracted in the EFA. Participants attributed high importance to both of them. Overall positive experiences were reported only for PCC. The delivery of health services received a poor evaluation. Significantly worse experiences were observed for women and people that were either older, unemployed, with comorbidities or lower self-reported health, or less engaged in their healthcare management.
Spiers et al, 2015 [68]; UK	To identify outcomes identified as being important to people with LTNCs using integrated health and social care.	Secondary care; Neurological care	Qualitative: interviews	35 people with LTNCs.	Integration is an 'organising principle for care delivery that aims to improve patient care' (61, p.3].	20 outcomes were identified across three domains: personal comfort outcomes, social and economic participation outcomes, and autonomy outcomes. Inter-relationships between outcomes, both within and across domains, were evident.
Spoorenb	To evaluate the opinions and	Secondary	Qualitative:	23 older	None provided.	Participants' responses concerned firstly experiences with aging, including struggling with health; increasing dependency;

erg et al, 2015 [71]; Netherlands	experiences of community-living older adults with regard to integrated care and support.	care; Community care, care for older people, chronic care	interviews	adults with chronic conditions.		decreasing social interaction; loss of control; and Fears. Secondly, participants' experiences with Embrace included relationship with the case manager; interactions; and feeling in control, safe, and secure. The prospect of becoming dependent and losing control was a key concept in the lives of the older adults. Embrace reinforced participants' ability to stay in control, even if they were dependent on others. Participants felt safe and secure, in contrast to the fears of increasing dependency within the standard care system.
Stoop et al, 2020 [77]; International (Europe)	To provide insight at an overarching level, into activities aimed at improving person-centredness within integrated care sites.	Secondary care; Care for older people, home care	Mixed methods: multiple embedded case study design	244 older people.	Integrated care is defined as those approaches that proactively seek to structure and coordinate health and social care for older people in their home environments, centred around older people's needs [13].	Experiences of service users were mixed. For some activities (e.g. enablement services), discrepancies were identified between the views of service providers and service users.
Stray et al, 2024 [94]; International (global)	To explore how home-dwelling older adults experience communication within integrated care.	Primary and secondary care Home care, care for older people	Meta-ethnographic review	Older adults.	Integrated care is 'an organising principle for care delivery that aims to improve patient care and experience through improved coordination' [61, p.3].	Older adults are aware of IPC and have preferences regarding how it is conducted. Inconsistent care was perceived as a lack of IPC. There were varying preferences regarding involvement and awareness of IPC. A lack of IPC may trigger negative feelings.
Tuzzio et al, 2021 [90]; US	To identify opportunities to align care with the personal values of patients with complex medical, behavioral, and social needs.	Primary care; Complex care, care for older people; integrated health and social care;	Qualitative: Interviews	24 patients with complex care needs.	None provided	Findings included the importance of care teams exploring and acknowledging a patient's values, providing access to nonphysician providers who have different perspectives on care delivery, offering values-aligned mental health care, ensuring connection to community-based resources that support values and address needs, and providing care that supports the patient and their family and caregivers.

		opioid use; obesity; mental health				
Van Overveld et al, 2017 [76]; Netherlands	To explore needs and preferences of patients with head and neck cancer (HNC), to adapt current integrated care to be more patient-centred.	Secondary and tertiary care; Cancer care	Qualitative: Interviews	14 participants: 12 patients and 2 chairmen of patient associations.	None provided	Findings included the personalisation of health care regarding patient values; clear insight into the healthcare process at organisational level; use of personalised communication, education and information that meets patients' requirements; adequate involvement of allied HCPs for physical support; more attention to the impact of HNC and its treatment; adequate involvement of family and friends; adequate general practitioner involvement in the aftercare; and waiting time reduction.
Walker et al, 2016 [89]; US	To describe the development and testing of a multidimensional self-report measure of patients' experiences of integrated care	All care settings; Integrated care	Quantitative: Cross-sectional survey	317 adults who had used healthcare at least twice in past 12 months.	Integrated care can be defined as 'a sense of "cohesiveness and connectedness of the health care system' [4]. Integrated care is 'patient care that is coordinated across professionals, facilities, and support systems; continuous over time and between visits; tailored to the patients' needs and preferences; and based on shared responsibility between patient and caregivers for optimizing health' [60, p.113].	Scales measuring five domains were confirmed: coordination within and between care teams, navigation (arranging appointments and visits), communication between specialist and primary care doctor, and communication between primary care doctor and specialist. Four of these demonstrated excellent internal consistency reliability. Mean scale scores indicated low levels of integration.
Weiss et al, 2022 [75]; US	To describe patient and provider experience with New York State PCMH and its key components	Primary care; Integrated care; patient-centred care	Qualitative: interviews and focus groups.	77 patients.	None provided	There was evident progress and some satisfaction with the PCMH model, particularly regarding integrated behavioural health and, to some extent, expanded use of electronic health records. There was less evident progress regarding improved access and reasonable wait times, which caused patients to continue to use urgent care or the emergency department as substitutes for primary care.

Zulman et al, 2017 [86]; US	To evaluate how augmenting the Veterans Affairs (VA) medical home (Patient Aligned Care Teams [PACT]) with an Intensive Management program (ImPACT) influences high-need patients' costs, health care utilisation, and experience	Primary care; Veteran care; complex care	Quantitative : RCT.	583 outpatients whose health care costs or hospitalisation risk were in the top 5% for the facility.	None provided	There were similar decreases in expenditure and acute and extended care utilization rates. 96% reported that they would recommend the ImPACT programme to others and there were modest increases in satisfaction with VA care.
